# A systematic literature review of schistosomiasis in urban and peri-urban settings

**DOI:** 10.1371/journal.pntd.0008995

**Published:** 2021-02-25

**Authors:** Katharina Klohe, Benjamin G. Koudou, Alan Fenwick, Fiona Fleming, Amadou Garba, Anouk Gouvras, Emma M. Harding-Esch, Stefanie Knopp, David Molyneux, Susan D’Souza, Jürg Utzinger, Penelope Vounatsou, Johannes Waltz, Yaobi Zhang, David Rollinson

**Affiliations:** 1 Global Schistosomiasis Alliance, London, United Kingdom; 2 Centre Suisse de Recherches Scientifiques en Côte d’Ivoire, Abidjan, Côte d’Ivoire; 3 Imperial College, London, United Kingdom; 4 Schistosomiasis Control Initiative Foundation, London, United Kingdom; 5 World Health Organization, Geneva, Switzerland; 6 Clinical Research Department, London School of Hygiene and Tropical Medicine, London, United Kingdom; 7 Swiss Tropical and Public Health Institute, Basel, Switzerland; 8 University of Basel, Basel, Switzerland; 9 Department of Tropical Disease Biology, Liverpool School of Tropical Medicine, Liverpool, United Kingdom; 10 Sightsavers, Haywards Heath, United Kingdom; 11 Helen Keller International, Regional Office for Africa, Dakar, Senegal; University of Cambridge, UNITED KINGDOM

## Abstract

**Background:**

Schistosomiasis is a parasitic disease caused by trematode worms of the genus *Schistosoma* and belongs to the neglected tropical diseases. The disease has been reported in 78 countries, with around 290.8 million people in need of treatment in 2018. Schistosomiasis is predominantly considered a rural disease with a subsequent focus of research and control activities in rural settings. Over the past decades, occurrence and even expansion of schistosomiasis foci in peri-urban and urban settings have increasingly been observed. Rural–urban migration in low- and middle-income countries and subsequent rapid and unplanned urbanization are thought to explain these observations. Fifty-five percent (55%) of the world population is already estimated to live in urban areas, with a projected increase to 68% by 2050. In light of rapid urbanization and the efforts to control morbidity and ultimately achieve elimination of schistosomiasis, it is important to deepen our understanding of the occurrence, prevalence, and transmission of schistosomiasis in urban and peri-urban settings. A systematic literature review looking at urban and peri-urban schistosomiasis was therefore carried out as a first step to address the research and mapping gap.

**Methodology:**

Following the Preferred Reporting Items for Systematic Reviews and Meta-Analyses (PRISMA) guidelines, a systematic computer-aided literature review was carried out using PubMed, ScienceDirect, and the World Health Organization Database in November 2019, which was updated in March 2020. Only papers for which at least the abstract was available in English were used. Relevant publications were screened, duplicates were removed, guidelines for eligibility were applied, and eligible studies were reviewed. Studies looking at human *Schistosoma* infections, prevalence, and intensity of infection in urban and peri-urban settings were included as well as those focusing on the intermediate host snails.

**Principal findings:**

A total of 248 publications met the inclusion criteria. The selected studies confirm that schistosomiasis is prevalent in peri-urban and urban areas in the countries assessed. Earlier studies report higher prevalence levels in urban settings compared to data extracted from more recent publications, yet the challenge of migration, rapid uncontrolled urbanization, and resulting poor living conditions highlight the potential for continuous or even newly established transmission to take place.

**Conclusions:**

The review indicates that schistosomiasis has long existed in urban and peri-urban areas and remains a public health problem. There is, however, a challenge of comparability of settings due to the lack of a clear definition of what constitutes urban and peri-urban. There is a pressing need for improved monitoring of schistosomiasis in urban communities and consideration of treatment strategies.

## Author summary

Schistosomiasis is an infectious parasitic disease and one of the 20 diseases considered by the World Health Organization as a neglected tropical disease. It is typically associated with poor environmental and sanitary conditions, primarily affecting rural communities. However, with the currently observed rapid urbanization and predicted two-thirds of the world population living in urban areas by 2050, concerns are raised about a spread of schistosomiasis to urban and peri-urban areas. At the same time, there seems to be little knowledge of the extent of schistososomiasis in urban areas. In light of the efforts to control morbidity and ultimately achieve elimination of schistosomiasis as well as reaching Sustainable Development Goals 3 “achieve health for all” and 11 “make human settlements inclusive, safe, resilient, and sustainable,” this systematic literature review was conducted to address the existing research and mapping gaps and to contribute to the understanding of the burden of schistosomiasis in areas that have not, as yet, been the focus of control efforts and mass drug administration programs. The review suggests that rapid and unorganized urbanization and resulting poor living conditions in urban and peri-urban areas may lead to new disease foci and thereby increase the overall disease burden. Research and policy implications are discussed.

## Introduction

Schistosomiasis is a parasitic disease caused by trematode worms of the genus *Schistosoma*, from which the disease derives its name. Schistosomiasis is one of the 20 diseases considered by the World Health Organization (WHO) as neglected tropical diseases (NTDs). The disease has been reported in 78 countries, 52 of which have moderate to high transmission requiring large-scale intervention through preventive chemotherapy [1]. Praziquantel is the recommended drug for treatment and WHO estimated that around 290.8 million people were in need of treatment in 2018 [1].

The infective larvae (cercariae) develop within freshwater snails and when released penetrate the skin of a human/mammalian body in contact with the water. The adult worms reside within blood vessels of the human host, and eggs are expelled in urine (*S. haematobium*) or feces (*S. mansoni*, *S. japonicum*, *S. mekongi*, *S. intercalatum*, and *S. guineensis*). In both rural and urban areas with poor sanitation, this may result in eggs reaching open freshwater bodies, including man-made sources such as irrigation canals and dams, facilitating infection of snails and the continuation of the life cycle [2].

There are two forms of the disease: (i) intestinal schistosomiasis, which results in abdominal pain, diarrhea, blood in the stool, and in advanced stages also leads to the enlargement of liver and spleen, liver fibrosis, portal hypertension, and accumulation of fluid in the peritoneal cavity; and (ii) urogenital schistosomiasis, which results in blood in urine, pain during micturition, and in advanced stages also leads to fibrosis of bladder and ureter, as well as kidney damage and bladder cancer [2]. Genital forms can manifest in pain of the testicle and blood in the sperm in men [3]. In women, eggs deposited in the tissues of the cervix and lower female genital tract lead to intravaginal lesions resulting in genital itching, pain, bleeding as well as dyspareunia. Moreover, eggs which deposited in the uterus and fallopian tubes can lead to infertility. In particular, female genital schistosomiasis is known to be an important HIV/AIDS cofactor in co-endemic areas [4].

Prevalence and transmission of the different types of schistosomiasis are typically associated with poor environmental and sanitary conditions, thus usually affecting people living in unfavorable socioeconomic conditions [5,6]. Due to environmental degradation and climate change, leading to warmer, tropical temperatures, and shifting rainfall patterns, there may be a change and even increase in the geographic and periodic distribution of *Schistosoma* spp. infections [5,7]. Hotez [8] draws attention to “new global hot zones for emerging and parasitic diseases” due to combined forces of climate change, urbanization, and poverty, possibly explaining why we are already seeing emergences of NTDs in wealthy G20 countries, for example, urogenital schistosomiasis in Corsica, France [8,9].

Schistosomiasis is predominantly considered a rural disease, associated with places where people are reliant on natural freshwater bodies for everyday activities, with a subsequent focus of research and control activities in rural settings. Yet, over the past decades, occurrence and even expansion of schistosomiasis foci in peri-urban and urban settings have increasingly been observed [9–12]. Rural–urban migration in low- and middle-income countries (LMICs) and subsequent rapid and unplanned urbanization are thought to explain these observations [13]. Fifty-five percent (55%) of the world population is already estimated to live in urban areas, with a projected increase to 68% by 2050 [14]. Moreover, 90% of this increase in urban populations is projected to take place in Africa and Asia [14], including countries affected most by schistosomiasis. Such rapid urbanization is known to lead to informal settlements with low-quality housing, poor sanitation, and waste management as well as limited availability of, and access to, formal health services with subsequent health risks and social inequities [15,16]. The risk of transmission of infectious diseases, including schistosomiasis, is thereby amplified.

It is important to note that estimations of the rate of urbanization and the number of people living in urban areas vary since there is no universal definition of “urban” and “peri-urban” [17]. According to the Food and Agricultural Organization (FAO) of the United Nations, “because of national differences in the characteristics that distinguish urban from rural areas, the distinction between urban and rural population is not amenable to a single definition that would be applicable to all countries. Rural areas are usually defined as ‘what is not urban’ (UN, 1998, 2004), and so inconsistencies in the definition of what is urban lead to inconsistencies in characterizing what is rural” [18].

In light of rapid urbanization and the efforts to control morbidity and ultimately achieve elimination of schistosomiasis, it is important to have a sound understanding of the occurrence, prevalence, and transmission of the disease in urban and peri-urban settings. However, according to unpublished country experiences [19] and national programs, there is uncertainty as to whether peri-urban and urban areas are included in schistosomiasis mapping activities and national control and elimination efforts. Therefore, as countries intensify control and elimination interventions, it is necessary to identify research and mapping gaps and ensure accurate estimates of schistosomiasis prevalence in countries in order to develop protocols for control and elimination interventions in urban and peri-urban settings. As a first step to address the research and mapping gap, a systematic literature review was conducted.

## Methods

### Protocol and registration

A review protocol was written and registered with PROSPERO (https://www.crd.york.ac.uk/prospero/display_record.php?ID=CRD42020164873 or registration number: CRD42020164873).

### Definition of urban and peri-urban

As there are no clear definitions for urban and peri-urban areas, schistosomiasis research studies referring to urban and peri-urban settings, towns, and cities were therefore included without further inquiry. In the context of Chinese publications, insight from Chinese colleagues was sought to better understand the administrative levels. When neither abstracts nor further research into the study area provided clarity as to the nature of the setting, the paper was not included in the review.

### Eligibility criteria

All studies looking at human *Schistosoma* infections, prevalence, and intensity of infection in urban and peri-urban settings were included. Similarly, studies focusing on intermediate host snails for human schistosomiasis in urban and peri-urban areas were included. There were no restrictions as to geography or age or sex of the people assessed. The search excluded studies that looked at nonhuman infection with *Schistosoma* other than snails. Furthermore, publications looking at urban residents becoming infected as tourists in rural areas were not included when they did not also assess prevalence levels in specific urban or peri-urban areas more generally [20].

### Information sources and search

A systematic computer-aided literature review was carried out using PubMed, ScienceDirect, and WHO Database. The search was conducted in November 2019 and updated again in March 2020. The following search terms were utilized: “urban schistosomiasis,” “urban AND schistosomiasis,” urban AND schisto*[title], urban[title] AND schisto*[title], urban AND schisto*[title/abstract], urban[keyword] AND schisto*[keyword]. The above search terms were also employed, using peri-urban, town, towns, city, and cities, instead of urban.

No further selection was made according to study design or countries of study. The methodologic quality of the individual studies was not assessed. No differentiation was made between the different forms of schistosomiasis. In order to obtain an understanding as clear as possible of the research carried out into urban schistosomiasis, all available records were considered for eligibility for the review, with data from 1953 through to March 2020 included. The cutoff point was March 25, 2020.

### Study selection

**Fig 1:**
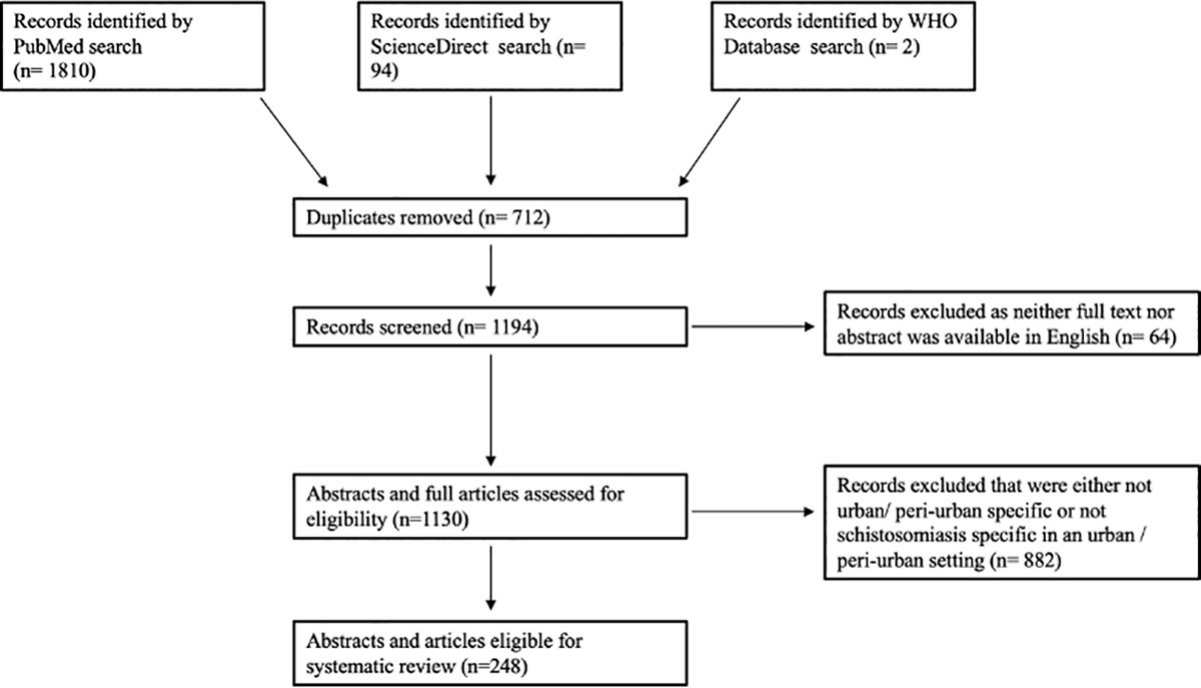
Selection criteria and process used in this review.

The study selection process is outlined in Fig 1. Only studies for which either the full paper or the abstract were available were considered in the analysis. Studies written in a language other than English were included, provided the abstract was in English, meaning that only data extracted from the English abstract were included and no data were extracted from any non-English text. The quality of the underlying data of the publications was not assessed. Moreover, studies for which there was no information about a proceeding peer-review process were also included. As mentioned, the quality of the data was not assessed, but an extensive inclusion of studies was aimed for.

### Data items

Data on prevalence and intensity of infection in humans were of importance for the review, as well as malacologic data, i.e., prevalence and density of both infected and noninfected intermediate host snails. Furthermore, information on location, demographics as well as the year of data collection were recorded where available. Most studies were cross-sectional. Where possible, data shared in the papers on infection over time were extracted and reported on in the review in order to establish an understanding of how urban schistosomiasis has changed in that particular setting over time. Furthermore, information on factors identified or assumed to be of relevance for driving transmission of *Schistosoma* infections in urban and peri-urban settings was recorded. Details can be found in S1 Table.

### Risk of bias in individual studies and risk of bias across studies

In this review of available studies on urban schistosomiasis, the methodological quality of the individual studies was not assessed. This may have increased the risk that biased papers were included. However, since the purpose of the review was to determine whether there is evidence of urban schistosomiasis, having a greater number of studies included in the review was considered beneficial. The next step, if there is evidence suggestive of urban schistosomiasis, is to conduct high-quality prevalence surveys to have more accurate prevalence estimates. The main criterion was whether the studies took place in urban and/or peri-urban settings. Since only publications available in English or with an English abstract were included, the review has a bias toward publications in this language.

### Synthesis of results

Recurring themes were summarized via a combination of thematic synthesis and textual narrative synthesis, meaning that descriptive themes were generated following the analysis of the papers, such as “migration,” “sanitary infrastructure,” and “awareness.” Findings are organized according to the themes identified, based on word analysis, not quantitative analysis of numbers presented in the papers. Numeric data are then presented within the themes. Exceptions and outliers were equally addressed in order to fully capture the findings of urban schistosomiasis so far.

## Results

**Fig 2:**
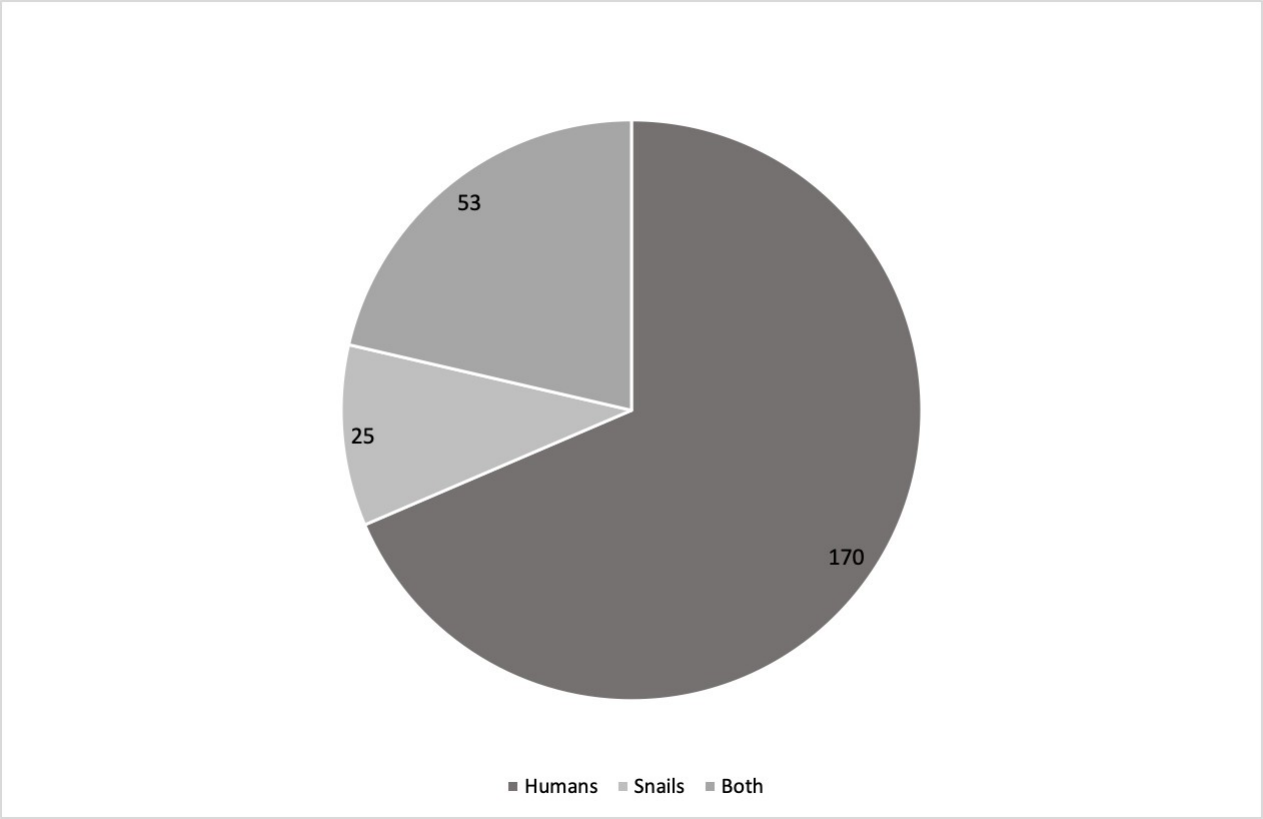
Number of studies looking at humans only, snails only, or both.

The selection process and results obtained are shown in Figs 1 and 2 and Table 1.

**Table 1:** Year, country, and number of published papers reporting on urban schistosomiasis included in our review, stratified by geographic region.

Year	Africa		Asia		Latin and Central America		Middle East and North Africa		Other	
*1953–1978*										
	Libya	1	Japan	1	Brazil	2	Egypt	1	USA	2
	Madagascar	1	Philippines	2						
	Mauritania	1								
	Mauritius	1								
	Nigeria	1								
	Zambia	1								
*1979–1988*										
	Cameroon	3			Brazil	3			N/A	1
	Central African Republic	1			Dominican Republic	2				
	Ethiopia	1								
	Liberia	1								
	Republic of the Congo	1								
	Tanzania	2								
	Zimbabwe	1								
*1989–1998*										
	Botswana	1	China	1	Brazil	24	Egypt	2	N/A	1
	Burundi	2			Dominican Republic	1	Yemen	1		
	Cameroon	1			Venezuela	1				
	Côte d’Ivoire	1								
	Equatorial Guinea	2								
	Ethiopia	8								
	Kenya	1								
	Libya	1								
	Mali	1								
	Niger	1								
	Nigeria	5								
	Republic of Sao Tome and Principe	2								
	Senegal	4								
	Tanzania	2								
	Uganda	2								
	Zambia	2								
	Zanzibar	1								
	Zimbabwe	1								
*1999–2008*										
	Burkina Faso		Cambodia	1	Brazil	19	Egypt	2		
	Cameroon	3	China	1			Yemen			
	Côte d’Ivoire	2								
	Ethiopia	1								
	Madagascar	1								
	Mali	1								
	Niger	3								
	Nigeria	8								
	Tanzania	1								
	Zimbabwe	4								
*2009–2018*										
	Angola	1	China	16	Brazil	24	Egypt	2		
	Benin	1					Yemen	1		
	Côte d’Ivoire	4								
	Democratic Republic of the Congo	1								
	Ethiopia	15								
	Ghana	2								
	Kenya	4								
	Mali	2								
	Malawi	1								
	Mauretania	1								
	Mozambique	1								
	Nigeria	7								
	Sudan	2								
	Tanzania	3								
	Uganda	2								
	Zambia	1								
	Zanzibar	1								
*2019–2020*										
	Ethiopia	3	China	2	Brazil	2				
	Ghana	1	Philippines	1						
	Uganda	1								

A table of all papers included in this review is provided in S1 Table.

As shown in Table 1, publications on urban schistosomiasis date back to the 1950s. With increasing urbanization, the number of publications on urban schistosomiasis published per year also increased. Table 1 also shows that the majority of publications on urban schistosomiasis came from Brazil (*n* = 76) and Ethiopia (*n* = 27), followed by China (*n* = 21) and Nigeria (*n* = 21).

The majority of the studies set out to study urban schistosomiasis either in comparison to rural schistosomiasis, to assess changes over time, or to specifically assess prevalence and intensity levels in certain areas and among certain population groups at that point in time [21–24]. Many studies also included questionnaires in order to better understand the socioeconomic circumstances of the study participants, as well as to learn more about their habits regarding their water-related recreational activities, source and use of water, as well as access to sanitary facilities [25–27].

The selected studies confirm that schistosomiasis is or was prevalent in peri-urban and urban areas in the countries assessed. Point prevalence estimates ranged from low (below 1% in Tanga, Tanzania [13]; 1.9% in Korhogo, Côte d’Ivoire, 2015 [28]; 8.4% in Jimma Town, Ethiopia [29]) to moderate (17.4% in Ibadan, Nigeria [30]; 20.7%, Lusaka, Zambia, 2007 [31]; 24.9%, State of Alagoas, Brazil [32]) to high (61.5%, Adwa, Ethiopia [33]; 60.9%, Abeokuta, Nigeria in 1987 [34]]). Years of data collection have been provided when specifically indicated in the papers.

### Urban schistosomiasis—general findings

Overall, certain factors were found to be significantly associated with a lower or higher risk of infection in urban areas: Distance from the individuals home to a freshwater access point in which disease-relevant mollusc species were present [5], abstaining from or swimming in open freshwater bodies [28,35–37], as well as the profession of the parents, and socioeconomic status [38–40].

Similarly to findings from rural areas, across the studies reviewed, boys in urban areas as well as age cohorts between 8 and 15 years old were more likely to be infected and to exhibit more intense infections than girls or other age groups [5,28,30,38,41].

### Urban schistosomiasis over time

Looking at prevalence and intensity levels of schistosomiasis in one place over time can further help to deepen the understanding of whether and how numbers have been changing in urban areas over time and where appropriate data are collected develop an understanding of the factors that contributed to that change. Silva and colleagues, for example, compared prevalence levels of *S*. *mansoni* infection in the São Bartolomeu in Salvador, Brazil, in 2011 and 2015, by comparing a cohort that was enrolled in a schistosomiasis treatment program in 2011 to one that was not. In 2011, the overall prevalence of infection was above 20%, and by 2015, it had decreased to 6% (*p* <0.01; effect size 0.20; 95% CI 0.15 to 0.27). In addition, they observed improvements in sanitary facilities (defined as number of households having a connection to septic tank or sewer, otherwise toilet flushes to rivers or streams) and the frequency of household flooding in that neighborhood over time. The authors concluded that while treatment with praziquantel may have helped in the decline in prevalence, the biggest impact was attributable to the structural and sanitary changes in the community [42]. Similarly, in order to understand whether and how urban schistosomiasis had changed over time, a comparative cross-sectional study was carried out in the urban area of Alhandra, Brazil, comparing prevalence levels in 2010 to those found in 1979 [43]. Prevalence of infection was at 10% in 2010, compared to 47% in 1979. Furthermore, in 2010, 1% of the collected *Biomphalaria glabrata* intermediate host snail specimens produced cercariae, compared to 6% in 1979 (no information provided with regard to the statistical significance). The authors attributed these improvements to the mass treatment program among the population that had taken place between 1977 and 1979 and successive treatments of positive cases between 1982 and 2010, as well as sanitary improvements.

With many publications, however, it is not clear whether the prevalence levels reported and referred to are comparable. For example, a study carried out both in Dar es Salaam and Tanga in Tanzania compared the status of urogenital schistosomiasis among primary school children after a decade of anthelmintic treatment and found a low burden in both cities: 1.2% in Dar es Salaam and 0.3% in Tanga [13]. Previous studies had found prevalence levels in Dar es Salaam of 5% to 55% [41], 68% [44], and 48% [45] and in Tanga of 16% to 32% [46]. In addition to available treatment programs that had taken place to varying degrees in both cities between 2001 and 2009 (Dar es Salaam) and 2010 (Tanga), fewer snail habitats as a result of environmental change as well as improved levels of hygiene were considered to have contributed to the decrease. Another study carried out in Dar es Salaam, in comparison, looked at preschool-age children and found a *Schistosoma* spp. infection prevalence of 16% [47]. Different methodologies and diagnostics used are likely to explain some of the differences. Said and colleagues [47], for example, employed point-of-care circulating cathodic antigen (POC-CCA), urine filtration, Kato-Katz, FLOTAC, and Baermann tests to detect helminth infections in urine and stool, while Mwakitalu and colleagues [13] assessed for eggs in urine and stool using urine filtration and Kato-Katz techniques only.

Overall, when looking at prevalence in studies conducted in the same place at different time points, prevalence tend to also indicate a decline, while still persisting [34,48–50], but it is difficult to draw direct comparisons.

### Explanation for schistosomiasis prevalence in urban areas

There were a number of recurring themes used to explain the prevalence of *Schistosoma* infection and risk for further establishment of disease foci in urban and peri-urban areas. These were mainly migration to urban areas and consequent unorganized urbanization and environmental degradation, a lack of safe water, sanitation, and hygiene (WASH) facilities as well as the absence of awareness for the disease among the urban communities [51,52].

#### Urbanization and lack of sanitary infrastructure

While urbanization per se was not highlighted as influencing prevalence of urban schistosomiasis, proximity of water sources, lack of adequate sanitary waste disposal, and living in poor shantytowns were recurring themes [16,39,53]. A study in Uganda, for example, assessed the relationship between exposure to wastewater and fecal sludge and schistosome infection using demographic and socioeconomic data as well as stool samples analyses using the Kato-Katz technique. Urban farmers exhibited the highest point prevalence (23% for *S*. *mansoni*), compared to slum dwellers and workers collecting fecal sludge or maintaining drainage channels (5% or above; difference x^2^, *p* <0.001) [54]. A lack of sanitary infrastructure and clean water supports fecal contamination of natural aquatic environments in new urban areas. Intermediate host snails can thereby become infected, leading to new sources of transmission. In line with these findings, Gomes and colleagues [7] highlighted a direct relationship between the contamination of the environment with human waste and foci of active *Schistosoma* transmission.

#### Migration

The movement of people with active schistosome infections into urban areas creates possibilities for rapid introduction of transmission of the disease in places where the intermediate snail hosts exist [55–57]. A literature review by Kloos and colleagues [11] suggests the need for research into different types of migration patterns, in order to fully understand, forecast, and assess the risk of future spread of schistosomiasis. In addition to rural–urban migration, the authors mention the relevance of urban–urban, urban–rural movement as well as movements of environmental refugees and tourists as a result of social-economic dynamics for the spread of schistosomiasis. Feeding into this review, Kloos and colleagues [39] found labor migration patterns from, and periodic returns to, rural areas to be associated with *Schistosoma* infections. Other studies, however, did not show such clear relationships between migration and presence of infection [50,58].

#### Presence of snails and environmental changes

A range of studies carried out malacologic surveys, including parasitologic analysis of the molluscs. In particular, studies conducted in Brazil paid attention to the intermediate host snail species [59]. The high abundance of cercaria-shedding snails as well as irrigation activities in peri-urban and urban areas suggested the need for focal snail control in prospective control interventions that accompany treatment programs [60,61].

Moreover, one study assessed the influence of rainfall seasonality on prevalence of infection among school-age children in a town in southern Mauritania. It was observed that dry season, meaning the months during which there is a lack of precipitation, appeared to increase the risk for *S*. *haematobium* infection [62]. Such findings suggest that environmental and climate changes that change rainfall patterns and temperature may influence the prevalence of schistosomiasis and thus the risk for the health of residents as well as that this influence may be different for urban and rural areas [63].

In particular, malacologic surveys appeared essential in understanding the prevalence of disease-relevant snail species, their infection levels, and changes in their distribution, with many of these studies having been carried out in Brazil [7,64]. The authors mentioned, for example, the disturbance and degradation of natural areas neighboring residential zones as responsible for the proliferation of molluscs. The situation was worsened by subsequent contamination with human waste, as described above [16]. Studying rainfall patterns and abundance of *B*. *glabrata*, Oliveira and colleagues concluded that climate change and changes in frequency and intensity of rainfall likely interfered with the focus of urban schistosomiasis in the Aracaju metropolitan area, Brazil [65]. Similarly, researchers from China [66] assessed the effectiveness of habitat modification with regard to snail control. Between 2003 and 2011, they observed an average decline in snail density of 99.83% and a schistosome infection rate of 0.31% resulting from smoothing beaches, or lowering some and raising other parts of beaches as well as cultivation in the area.

#### Disease awareness

Limited research has been carried out into availability of health education among urban residents and levels of knowledge, awareness, and perceptions (KAP) of schistosomiasis in urban settings. Two studies looked at levels of awareness, including attitudes and health seeking behaviors, of urban communities around Lake Victoria compared to rural communities, as well as the feasibility of carrying out health education via community health workers [67,68]. Despite disease prevalence levels of 36%, there was little awareness for the disease. In one urban area in Brazil, it was found that illiterate individuals (30.1%) were at greater risk for periportal fibrosis. Both an education level of up to 11 years as well as specific prior treatment against schistosomiasis, suggesting an awareness for the disease, in turn, were found to be preventive factors for severe periportal fibrosis [69].

## Discussion

Schistosomiasis has been documented in urban and peri-urban areas across the world, with publications from Central and Latin America, sub-Saharan Africa, and Asia. Published studies date back to the 1950s; however, it seems that with an increasing rate of urbanization, an increasing number of publications report on this particular issue. Whether the proportion of studies on urban schistosomiasis to total studies reporting on schistosomiasis has changed was not analyzed.

Overall, when looking at prevalence in studies conducted in the same place at different time points, prevalence tend to indicate a decline, while still persisting [34,48–50], but it is difficult to point to a clear direction with regard to schistosomiasis infections for particular settings let alone for regions more generally. This is because (i) very few longitudinal studies were identified in the review; and (ii) the majority of publications looking at schistosomiasis prevalence over time refer to prevalence levels indicated in earlier publications or reports without necessarily commenting on the comparability of methodologies employed and thus on the comparability of results. Furthermore, the review suggests patchy availability of both up-to-date prevalence levels in individual countries and major urban and peri-urban areas in most or all endemic countries.

The review points to a number of recurring themes associated with schistosomiasis in urban and peri-urban areas. Studies continuously report on gender, i.e., being male, the age cohort 8–15 years, poor living conditions as well as the proximity to freshwater water bodies with intermediate host snails to all be associated with a higher risk of infection and more intense infections. Proximity to water includes housing, distance of respective schools to water bodies, as well as the profession (e.g., vehicle washers and sand harvesters) of individuals and the associated necessary water contact. Improvements in sanitary facilities, more built-up living conditions, active changes to landscapes (meaning fewer natural environments for snails to inhabit and continue the parasite’s life cycle), as well as regular treatments, were all mentioned as explanations for reductions in prevalence and intensity of infection. It was also reported, however, that despite schistosomiasis being prevalent in urban and peri-urban areas, there was little awareness and knowledge of the disease and its transmission dynamics among affected populations, in particular compared to that found in rural communities.

Interestingly, despite the overall trend of reduced prevalence levels in urban areas, authors continuously raised the challenge of migration, rapid uncontrolled urbanization (i.e., nonregulated growth of urban and peri-urban areas), as well as peri-urban gardens used for local food supply needs, to add to or exacerbate poor living conditions and to thereby increase the potential for continuous or even newly established transmission to take place. The importance of continued treatments, sanitary improvements, and education was therefore emphasized throughout in order to maintain the overall positive developments.

### Research and policy implications

#### Research implications

Five central research implications were identified: (i) the need for an operational definition of “urban” relevant to schistosomiasis; (ii) categorizing the urban and peri-urban environment with respect to transmission potential and risk of exposure; (iii) the need for a heightened awareness on the focality of infection, particularly relevant in urban settings; (iv) the need to assess where improved WASH and behavior change in the absence of mass drug administration (MDA) have led to a reduction in prevalence; and (v) the relationship between changes to the environment, the proliferation of molluscs, and the subsequent risk of transmission.

The lack of a definition of what constitutes a peri-urban and urban area makes comparability of research activities and findings difficult. While it may not be possible to come up with a universal definition, as outlined in the introduction, a description in published papers of the area studied could help to contextualize the findings and increase comparability of publications and can help to establish a definition relevant to schistosomiasis research. Furthermore, the definition and context of urban and peri-urban areas will have changed over time, which can make comparisons over time and in different settings more difficult.

While some studies mentioned and even discussed explanations for the prevalence of schistosomiasis in urban areas and how new urban schistosomiasis foci may become established, there were no publications that had studied these factors together with changes in prevalence rates over time in order to establish a clear correlation or causational effect. Studies do, however, indicate great variation in the risk for, and prevalence of, schistosomiasis across urban and peri-urban settings. Categorizing the urban and peri-urban environment according to infection already being prevalent as well as areas with a potential for transmission and the respective reasons can provide helpful information on the focality of infection, which, in turn, can feed into the development of setting-specific elimination strategies. This requires more systematic mapping of *Schistosoma* spp. prevalence and intensity among urban populations over time.

This is directly linked to the need to better understand the relationship between environmental changes due to urbanization, natural degradation as well as climate change, improved levels of hygiene, and both the occurrence of the intermediate snail hosts as well as their infection levels. Operational research looking into which environment changing factors and activities increase and which decrease the distribution of molluscs and subsequently the risk of infection can help to identify preventative measures that can feed into urban planning. Together with a better understanding of how nonregulated establishments of peri-urban gardens increase the risk of transmission, such research would be an important contributor to SDG 11 aimed to “make cities and human settlements inclusive, safe, resilient, and sustainable.”

#### Policy implications

The recurring theme of observations of a relationship between human waste disposal and the presence of active infection has immediate policy implications and can be a useful starting point for a risk assessment in peri-urban and urban areas for the health of surrounding residents. With two-thirds of the world population expected to be living in megacities by 2050, and many of those megacities being characterized by unorganized and nonregulated settlements and a lack of municipal oversight and infrastructure provision, protocols for the provision of healthcare interventions targeting NTDs, including schistosomiasis, will be needed. This does, however, call for improved coordination with the WASH as well as the urban development sector and the need for heightened awareness of the problem.

Such healthcare interventions will have to pay particular attention to the heterogeneity of the urban population in terms of socioeconomic status with respective living conditions, accessibility of communities, and the challenges related to engaging with diverse groups of people, to name but a few [19]. In addition, assessments into whether healthcare facilities can detect infection adequately and respond to the health needs of the urban poor in particular may help efforts to optimize the delivery of MDA and supplement schistosomiasis mapping efforts, which, in the specific context of the urban environment, are prone to becoming outdated or being at a spatial resolution that does not adequately capture the focality of schistosomiasis.

Furthermore, the many studies reporting on intermediate host snail species shedding cercariae suggest the need to incorporate snail control in addition to preventive chemotherapy in order to address the health risks for urban populations. All the while, environmental consequences of such snail control need to be carefully assessed and considered, in particular in peri-urban and urban farm settings.

### Strengths and weaknesses of the present review

The present literature review intended to provide an overview of the research and survey activities carried out so far until early 2020 in terms of peri-urban and urban schistosomiasis. By including papers for which only the abstract was available, this review can give a more all-encompassing overview than would otherwise have been possible. The same applies to the time frame applied in the search, going back several decades to better understand changes over time.

At the same time, the scope of the review could have been improved by identifying and including additional studies by means of a snowballing search, i.e., including papers provided as references in the studies identified by the online search. Some papers may also have been missed out that do not mention urban, peri-urban, town(s), or city(ies) in the title, abstract, or keywords but provide information on rural–urban differences in text. While the keywords applied attempted to avoid this, this problem may not be entirely discarded. Similarly, while spot checks for megacities were carried out within the search, more specific literature review for megacities could be carried out in a second step. Furthermore, because publications in a language other than English (for which not even the abstract was available in English) were not included, the review is likely missing some studies. Finally, because papers were included for which only abstracts were available, no assessment could be made with regard to the quality of the methodology employed and the subsequent data published. Caution must therefore be taken when drawing conclusions from the data presented.

## Conclusions

The present systematic literature review has shown that schistosomiasis has been prevalent in urban and peri-urban areas for some time. Urban schistosomiasis is therefore not a recent phenomenon due to evolving and increasing urbanization or environmental degradation. The review suggests that overall, there has been a decline in schistosomiasis in urban and peri-urban areas over time, and the dynamic has been very different for every peri-urban and urban setting studied. Despite this decline, the studies included in the review mentioned a number of factors and determinants in their explanations for the continued presence of *Schistosoma* infections and the risk for new schistosomiasis foci, including increased and unorganized urbanization, a lack of adequate sanitary facilities, as well as a lack of awareness of schistosomiasis in urban areas. This review identified a number of research and policy implications. There is a need for more systematic mapping of schistosomiasis in peri-urban and urban settings as well as operational definitions of “urban” and “peri-urban.” These will help to better draw comparisons of prevalence over time. Ensuing next steps include the need for health services to be adequately prepared to diagnose and treat schistosomiasis, as well as for the WASH and urban development sectors to work together to achieve SDG 3 (“health for all”) and SDG 11 (“sustainable cities and communities”).

**Key learning points**

- Schistosomiasis in urban and peri-urban areas is not a recent phenomenon—prevalence and intensity levels have declined in areas studied.
- Increased and unorganized urbanization, a lack of adequate sanitary facilities, and a lack of awareness of schistosomiasis in urban areas are important factors for new or continued risk of schistosomiasis transmission.
- Operational definitions of “urban” and “peri-urban” are needed to better draw comparisons of prevalence over time.
- More systematic mapping of schistosomiasis in peri-urban and urban settings is encouraged to support adequate provisions of health services and water, sanitation, and hygiene (WASH) facilities.

**Top five papers**

1. Hotez PJ. Human parasitology and parasitic diseases: heading towards 2050. Adv Parasitol. 2018;100:29–38. Epub 2018 May 14. doi: 10.1016/bs.apar.2018.03.002. PubMed PMID: 29753341.
2. Mwakitalu ME, Malecela MN, Mosha FW, Simonsen PE. Urban schistosomiasis and soil transmitted helminthiases in young school children in Dar es Salaam and Tanga, Tanzania, after a decade of anthelminthic intervention. Acta Trop. 2014;133:35–41. Epub 2014 Feb 6. doi: 10.1016/j.actatropica.2014.01.012. PubMed PMID: 24495630.
3. Adams AM, Vuckovic M, Birch E, Brant TA, Bialek S, Yoon D, et al. Eliminating neglected tropical diseases in urban areas: A review of challenges, strategies and research directions for successful mass drug administration. Trop Med Infect Dis. 2018;3(4). Epub 2018 Nov 25. doi: 10.3390/tropicalmed3040122. PubMed PMID: 30469342; PubMed Central PMCID: PMC6306919.
4. Silva LK, Barbosa LM, Kovach JD, Dos Santos Teixeira R, Soares ES, Cardoso CW, et al. The changing profile of schistosomiasis in a changing urban landscape. Int J Parasitol. 2020;50(1):27–34. Epub 2019 Nov 30. doi: 10.1016/j.ijpara.2019.10.003. PubMed PMID: 31783024; PubMed Central PMCID: PMC6964254.
5. Barbosa CS, Araujo KC, Sevilla MA, Melo F, Gomes EC, Souza-Santos R. Current epidemiological status of schistosomiasis in the state of Pernambuco, Brazil. Mem Inst Oswaldo Cruz. 2010;105(4):549–54. Epub 2010 Aug 20. doi: 10.1590/s0074-02762010000400034. PubMed PMID: 20721507.

## Supporting information

S1 Table List of eligible papers with summary information on prevalence, sample population, and diagnostics employed. (XLSX)
